# Two-stage hepatectomy and associated liver partition and portal vein ligation for staged hepatectomy (ALPPS) in treating liver metastases of rectal cancer: a case report

**DOI:** 10.1186/s40064-015-0965-z

**Published:** 2015-04-22

**Authors:** Yusuke Ome, Kazuyuki Kawamoto, Tae Bum Park, Tadashi Ito, Keizo Ogasahara

**Affiliations:** Department of Surgery, Kurashiki Central Hospital, 1-1-1 Miwa, Kurashiki City, 710-8602 Okayama Japan

**Keywords:** Colorectal cancer, Liver metastasis, Two-stage hepatectomy, Associated liver partition and portal vein ligation, ALPPS

## Abstract

**Introduction:**

An innovative approach, called associated liver partition and portal vein ligation for staged hepatectomy(ALPPS), has made possible a marked increase in future liver remnant (FLR) volume over a short period of time, thus permitting extended hepatectomy.

**Case description:**

This report describes ALPPS in a 63-year-old male patient with rectal cancer and synchronous multiple liver metastases. The primary lesion was resected, followed by chemotherapy. We had planned to completely resect the metastases in both liver lobes, but CT volumetry revealed a very small FLR (364 ml, 29% of the total liver volume, 0.61% of total body weight). His indocyanine green retention rate at 15 minutes was 12.7%. Because of the risk of tumor progression in the interim, we performed ALPPS. During the first stage, the tumor in segment 3 was resected, the right lobe was mobilized, the liver was partitioned, and the right portal vein was ligated. The right hepatic artery, duct and vein were secured with vessel loops. CT on postoperative day 6 showed sufficient FLR increase (from 364 ml to 573 ml, or from 0.61% to 0.96% of total body weight) and ICGR15 improvement to 3.4%. The second stage of ALPPS was on postoperative day 7, completing resection of the metastases. The patient recovered well and was discharged 21 days after the second step.

**Discussion and evaluation:**

The ALPPS approach has many advantages, but it lacks evidence of long-term results. Considering the high mortality and morbidity rates of ALPPS, it is essential to evaluate its risks and benefits in individual patients and determine the strict criteria for this surgical method.

**Conclusions:**

ALPPS procedure rapidly increases FLR, permitting extended hepatectomy for patients with initially insufficient FLR.

## Introduction

Hepatectomy for colorectal liver metastases has proven effective and the only method that can result in radical cure (Rodgers & McCall 2000; Martin & Warren 2000; Penna & Nordlinger 2002). Future liver remnant (FLR) volume and liver function are important in preventing postoperative liver failure and determining the indication of surgery. In patients with metastatic liver tumors, the morbidity of viral hepatitis is lower than that of hepatocellular carcinoma. Many of these patients receive chemotherapy before hepatectomy, which may result in chemotherapy-induced liver damage. It is essential to maintain sufficient FLR volume in patients undergoing hepatectomy for liver metastases.

Patients with FLR volume inadequate for liver resection frequently undergo preoperative portal vein embolization (PVE) or two-stage hepatectomy often combined with PVE or portal vein ligation (PVL) (Azoulay et al. 2000; Adam et al. 2000; Jaeck et al. 2003; Jaeck et al. 2004). PVE, however, may not result in sufficient FLR hypertrophy, and tumors on the non-embolic side may rapidly progress. Because completion of resection requires a comparatively long period of time in both methods, residual tumors may be enlarged.

Associated liver partition and portal vein ligation (ALPPS) for staged hepatectomy was recently reported to be useful in patients with liver metastases and low FLR volume (Baumgart et al. 2011; de Santibañes & Clavien 2012; de Santibañes et al. 2012; Schnitzbauer et al. 2012; Li et al. 2013). This method has been found to markedly increase FLR volume over a short period of time and may overcome the problems of PVE or usual two-staged hepatectomy.

We describe a patient who underwent the ALPPS procedure for multiple liver metastases of rectal cancer and achieved R0 resection.

## Case report

**Figure 1:**
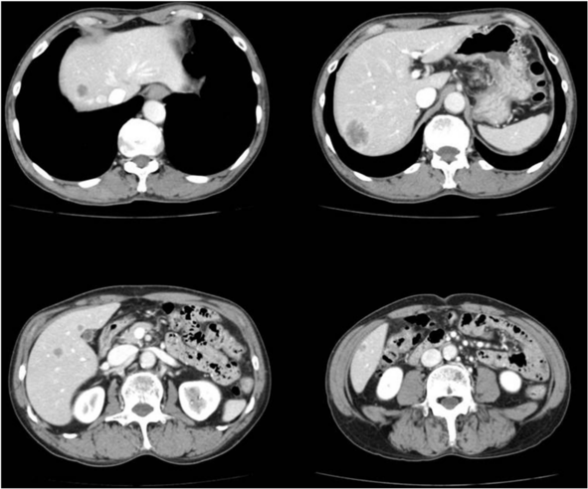
CT scan of this patient, showing multiple liver metastases in the liver.

A 63-year-old man presenting with a positive fecal occult blood test was diagnosed with middle rectal cancer by colonoscopy. He had a history of cerebral infarction and was taking an antiplatelet agent. Laboratory tests, including those for tumor markers, were approximately normal, and he was negative for markers of viral hepatitis. CT revealed synchronous multiple liver metastases, mainly in the right lobe (Figure 1). The patient was pathologically diagnosed with moderately differentiated tubular adenocarcinoma, classified as T3N1 in the TNM system. Because this patient had at least eight metastases in the liver, we decided to resect the primary lesion, by high anterior rectal resection in open sugery, followed by chemotherapy.

**Figure 2:**
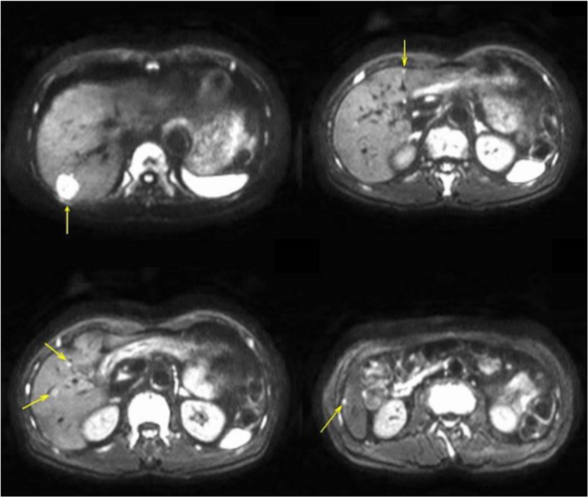
Diffusion weighted MRI of this patient, showing liver metastases in the right lobe and in segment 3.

Following resection of the primary tumor, the patient was started on postoperative chemotherapy with XELOX plus bevacizumab. CT showed a partial response after seven cycles of chemotherapy. However, the patient experienced side effects, including nausea, general fatigue, peripheral neuropathy, and anemia. He wanted to stop the chemotherapy and undergo liver resection. MRI revealed seven metastases in the right lobe, one in segments 4 and 5, and one in segment 3. R0 resection therefore required right hepatectomy and partial resection of segment 3 (Figure 2). His liver function tests were almost normal, but his indocyanine green retention rate at 15 minutes (ICGR15) was 12.7%, which was thought to be slightly deteriorated because of the chemotherapy. CT volumetry revealed a very small left lobe of the liver. The projected FLR volume was 364 ml, 29% of total liver volume and 0.61% of the total body weight that was 59.6 kg. The FLR volume would therefore be insufficient following resection. PVE was considered but might not always have induced sufficient remnant liver hypertrophy. Moreover, the time required to wait after PVE, about 4 weeks, may have increased the risk of tumor progression, especially on the non-embolic side. Conventional two-stage hepatectomy with PVE or PVL was also considered, but the appoach requires at least 4 weeks for complete resection after FLR hypertrophy, which develops adhesions. Furthermore, the first stage operation may further stimulate tumor growth due to the increase of release of growth factors. We therefore decided to adopt the ALPPS approach.

**Figure 3:**
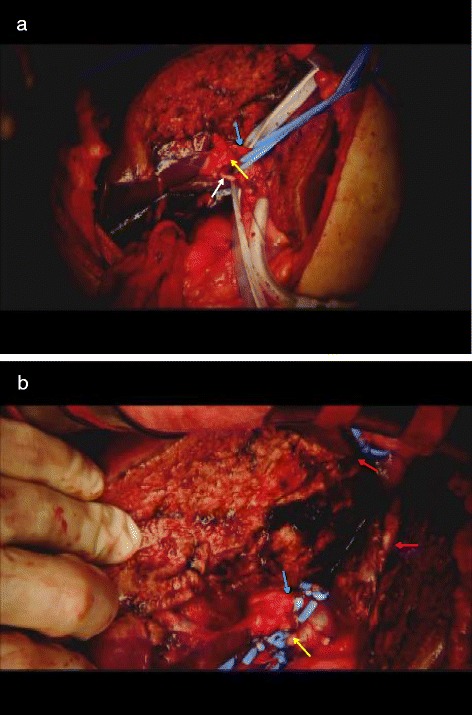
The first stage of ALPPS. **(a,b)** Shown are the right hepatic artery (yellow arrow), ligated right portal vein (white arrow), right hepatic duct (blue arrow), right hepatic vein (red arrow) and middle hepatic vein (purple arrow).

In the first stage of the ALPPS, we made a J-shaped incision, and ultrasonography confirmed that there were no other lesions. After mobilization of the right lobe, the gall bladder was resected and the right hepatic artery, right portal vein, and right hepatic vein secured with vessel loops. The tumor in segment 3 was enucleated, and the liver parenchyma was partitioned almost along the Rex-Cantlie line so that all other tumors were on the resected side. Total inflow occlusion using the Pringle maneuver was performed during parenchymal transection. The right hepatic duct was separated and secured with a vessel loop, followed by ligation of the right portal vein. The resulting partitioned right lobe retained artery inflow, biliary outflow, and hepatic outflow, but had no portal vein inflow (Figure 3). We did not use an exclusion bag for the right lobe specimen. A Blake drain® was inserted into the cut surface of the liver through the right subphrenic space. Seprafilm®, a bioresorbable membrane that prevents adhesion, was placed around the detached liver surface and under the abdominal incision. The operation required 216 minutes, and estimated blood loss was 798 ml. The total duration of the Pringle maneuver was 35 minutes.

**Figure 4:**
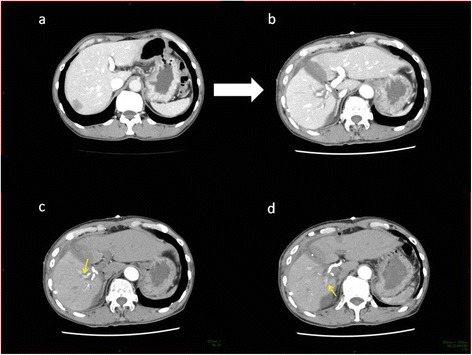
CT scan **(a)** before and **(b)** 6 days after the initial ALPPS, showing marked FLR enlargement. **(c,d)** arterial phase imaging. An arterioportal shunt was formed in the right Glissonian pedicle (yellow arrow).

The patient was able to ingest foods on the next day. He experienced only mild fatigue, but gradually recovered. Liver function tests peaked one day after surgery, with total bilirubin concentration at 1.8 mg/dl, AST at 1415 IU/l, ALT at 871 IU/l, LDH at 1091 IU/l and PT-INR at 1.17, with all showing gradual improvement. A CT scan on postoperative day 6 showed sufficient FLR enlargement, from 364 ml to 573 ml, or from 0.61% to 0.96% of total body weight (Figure 4). Growth rate was 34.8 ml/day. The partitioned right lobe atrophy was not seen, increasing from 889 ml to 908 ml. The right portal vein was occluded in the root, but intrahepatic portal vein flow was unexpectedly found in an arterial phase because of arterioportal shunt formation in the right Glissonian peddicle. ICGR15 was 3.4% on the same day.

The patient’s general condition and remnant liver function were thought to be sufficient for completion of resection. The second stage of ALPPS surgery was therefore performed on postoperative day 7.

**Figure 5:**
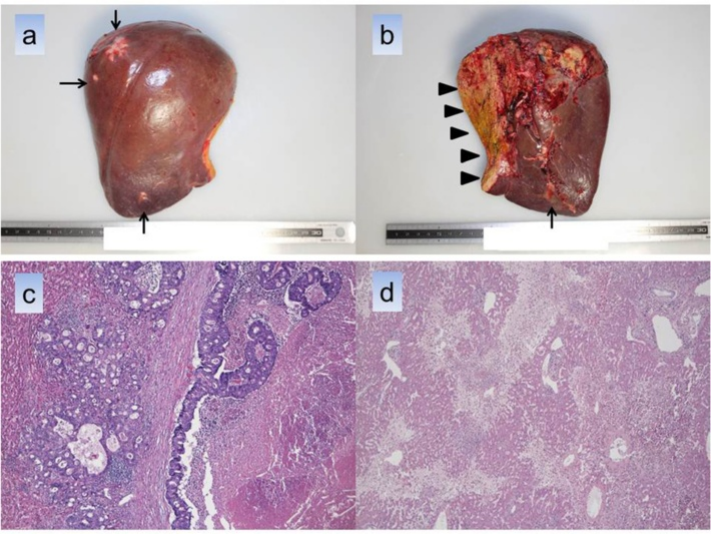
Histological findings of the right hepatectomy specimen. **(a,b)** multiple liver metastases (arrow) and cut surface (triangle). **(c)** tubular adenocarcinoma, **(d)** zone 3 (centrilobular) necrosis.

During the second stage operation, we made only a median incision. Although several adhesions were observed around the cut surface of the partitioned liver, all could be easily lysed. Both sides of the partitioned liver looked almost normal and viable. The vessels and the right hepatic duct secured during the first stage of ALPPS had been identified. The right portal vein and the right hepatic artery were ligated and cut. Then the right hepatic duct was divided with a stapler. The right hepatic vein was separated in the same way, followed by removal of the right hepatectomy specimen (Figure 5). The second stage operation required 52 minutes, and estimated blood loss was 160 ml. The patient required no blood transfusions during ALPPS.

Examination of the resected specimen showed multiple white solid nodules, of maximum size 27 × 25 mm. Some of the nodules were viable, and others were almost entirely necrotic because of preoperative chemotherapy. Macroscopic examination indicated the presence of hemorrhagic areas in some places. The multiple nodules were microscopically diagnosed as liver metastases from a known rectal adenocarcinoma (Figure 5b). Histological examination showed zone 3 (centrilobular) necrosis, a finding not observed in the adjacent (segment 3) specimen (Figure 5c). There was no evidence in this patient of sinusoidal dilatation caused by oxaliplatin-based chemotherapies.

**Figure 6:**
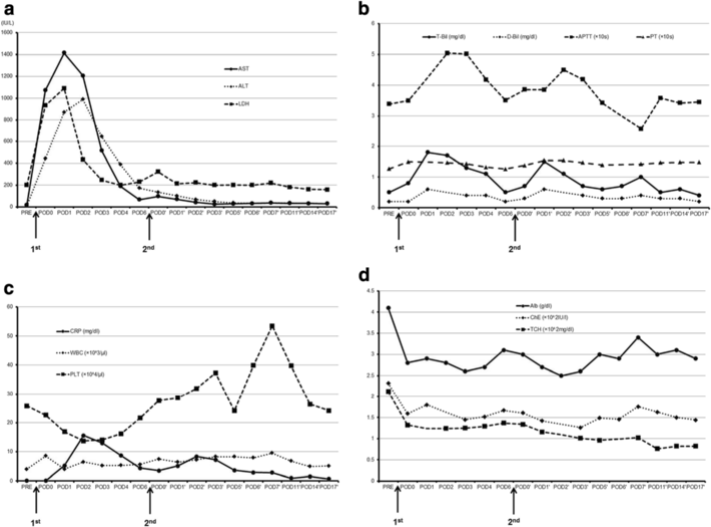
Perioperative laboratory tests. **(a)** AST, ALT and LDH. **(b)** T-Bil, D-Bil, APTT and PT. **(c)** CRP, WBC and PLT. **(d)** Alb, ChE and TCH.

The patient’s general condition improved markedly. Levels of transaminase and total bilirubin did not increase after the operation. Other laboratory tests showed nothing particularly abnormal (Figure 6). CT scans on postoperative day 7 showed greater FLR enlargement (from 573 ml to 643 ml), but the growth rate was slower than after the first stage operation (10 ml/day vs 34.8 ml/day). ICGR15 was 18.3% on postoperative day 14. This patient recovered uneventfully and was discharged home on postoperative day 21 after the second step. He now recieves adjuvant chemotherapy. He is free of tumor reccurence 5 months after ALPPS.

## Discussion

When extended hepatectomy is required for R0 resection, FLR volume may be sufficient or insufficient to prevent postoperative liver failure. If FLR volume is insufficient, PVE, PVL, or two-stage hepatectomy may be performed for FLR hypertrophy. FLR volume was thought to increase about 35% at 4 to 6 weeks after PVE (Hemming et al. 2003).A review article (Vyas et al. 2014) comparing PVE and PVL found that 4.8% of subjects in the PVE group and 7.4% of those in the PVL group failed liver hypertrophy, with 17.46% and 29.29%, respectively, showing interval disease progression. The resectability rate following PVE/PVL was estimated to be 78%. Moreover, patients with metastases on both lobes of the liver are at particularly great risk for a rapid increase of tumors in FLR after PVE/PVL (Hemming et al. 2003; Elias et al. 1999).

In conventional two-stage hepatectomy, minor resection of tumors on the FLR side often combined with first-stage PVE/PVL, followed by major extended lobectomy after about two months (Adam et al. 2000; Jaeck et al. 2003; Jaeck et al. 2004; Tanaka et al. 2007; Kianmanesh et al. 2008). This method was found to result in 30-40% FLR hypertrophy. However, these patients were also at risk of rapid tumor progression because the interval to radical resection is comparatively long. Resectability rates have been reported to range from 70-90%. PVE/PVL and hepatectomy may increase growth factors and cytokines, which may induce rapid tumor growth (Hemming et al. 2003; Elias et al. 1999; Kokudo et al. 2001; Jiang et al. 1999), Thus, the interval required for complete resection should be minimized.

The ALPPS procedure, first reported in 2011 (Baumgart et al. 2011), is an innovative staged hepatectomy that combines PVL and liver partition during the first stage of resection. Portal vein occlusion, interruption of intrahepatic vascular flow to the associated liver, and inflammation at the hepatectomy site have been found to promote FLR regeneration, as well as inducing rapid, marked hypertrophy. Using this method, FLR was reported to increase 70-90% in about a week (Schnitzbauer et al. 2012; Li et al. 2013; Alvarez et al. 2013; Torres et al. 2013; Schadde et al. 2014).

The partitioned liver, which is fed predominantly by the hepatic artery because of portal vein occlusion after the first stage operation, has been reported to complement liver functions until FLR enlargement (de Santibañes & Clavien 2012; de Santibañes et al. 2012). In this patient, an arterioportal shunt was formed in the Glissonean pedicle, recanalizing portal flow and suggesting that the partitioned liver had sufficient liver function. Thus, even if the second surgery had been postponed or canceled because of insufficient FLR hypertrophy, serious problems, such as liver insufficiency, necrosis, abscess, or biliary leak, would not have occurred.

We found that ICGR15 in this patient was markedly improved 6 days after the first stage operation. This was not only due to recovery from chemotherapy-induced liver damage, but to enlarged FLR and the activity of the remaining liver.

The rapid and marked hypertrophy resulting from ALPPS has many advantages. First, we usually perform minor hepatectomy, or clean-up resection on the non-PVE side, during the first stage. Second, the high rate of FLR increase makes possible extended liver resection in patients without sufficient FLR hypertrophy via the conventional approach. Third, the removal of residual tumors after a short interval can reduce the risk of disease progression, making the complete resection rate comparatively high, preventing the development of adhesions and facilitating the second step.

However, ALPPS has several drawbacks, including high operative mortality and morbidity rates. Severe complications, including liver failure, sepsis, and bile leakage, have been reported. Additionally, it is not clear whether increased liver capacity leads to functional improvement (Dokmak & Belghiti 2012). In performing right trisegmentectomy in ALPPS, both the arterial and portal vein flow to segment 4 are shut off after the first step, increasing the risk that segment 4 will become necrotic. However, as in this patient, ALPPS without the need to remove segment 4 (e.g. right hepatectomy) is comparatively safe. Our first experience with ALPPS showed an excellent short-term result.

The ALPPS approach is still a new surgical method, lacking evidence of long-term results. Complications, high risk groups and optimal candidates for surgery have not yet been identified. The high mortality and morbidity rates of ALPPS make it essential to evaluate its risks and benefits in individual patients and determine the strict criteria for this surgical method.

## Conclusion

Complete resection of liver metastases of colorectal carcinoma is the only potentially curative method. The ALPPS procedure induces FLR enlargement rapidly, permits extended hepatectomy and offers a chance of curative resection for patients with liver metastases.

## Consent

The patient provided written consent for publication of this report and any accompanying images.

## Abbreviations

ALPPS: Associated liver partition and portal vein ligation for staged hepatectomy
FLR: Future liver remnant
ICGR15: Indocyanine green retention rate at 15 minutes
PVE: Portal vein embolization
PVL: Portal vein ligation
CT: Computed tomography
MRI: Magnetic resonance imaging
